# The association between physical activity and body fat percentage with adjustment for body mass index among middle-aged adults: China health and nutrition survey in 2015

**DOI:** 10.1186/s12889-020-08832-0

**Published:** 2020-05-19

**Authors:** Qinpei Zou, Chang Su, Wenwen Du, Yifei Ouyang, Huijun Wang, Zhihong Wang, Gangqiang Ding, Bing Zhang

**Affiliations:** 1 National Institute for Nutrition and Health, Chinese Center for Disease Control and Prevention, Beijing, 100050 China; 2 Chongqing Center for Disease Control and Prevention, Chongqing, 400042 China

**Keywords:** Body fat percentage, Physical activity, Obese, Middle-aged adults

## Abstract

**Background:**

The inverse association between physical activity and body fat percentage (%) varies among different populations. We aim to examine whether the significant association between them was uniform across the subpopulations after taking into account body mass index (BMI).

**Methods:**

Our study relied on data from China Health and Nutrition Surveys in 2015, including 5763 participants aged 40–64 years from 15 regions. Physical activity was calculated as metabolic equivalent task hours per day (MET·h/d). Body fat% was measured by bioelectrical impedance analysis. Body mass index < 24 kg/m^2^ was defined as normal weight and BMI ≥ 24 kg/m^2^ was overweight/obese. The effects of physical activity on body fat% were estimated using the Kruskal-Wallis test among sex, age, BMI groups, education, income, region and urbanization. Quantile regression analyses were utilized to describe the relationship between physical activity and body fat% distribution.

**Results:**

Older adults, overweight/obese, higher education, higher income, residents of central China and those living in areas of higher urbanization had the lower physical activity. Participants who engaged in the highest level of physical activity had 2.0 and 1.5% lower body fat% than the lowest level of physical activity group (23.4, 34.8%) for men and women, respectively. There were 10.4 and 8.8% of normal weight males and females called normal weight obese. Overall, 1 h extra 4.5 MET•h/d was significantly associated with 0.079 and 0.110% less total body fat% at the 75th and 90th percentiles in normal weight males, with 0.071% less at the 25th percentiles in overweight/obese males, with 0.046–0.098% less at the 25th to 90th percentiles in normal weight females, and with 0.035–0.037% less from the 50th to 90th percentiles in overweight/obese females. The inverse association between physical activity and total body fat% was stronger in normal weight obese participants than other subgroups.

**Conclusions:**

In middle-aged Chinese adults, the inverse association between physical activity and body fat% was only in particular subpopulations rather than the entire population. We should pay much attention to normal weight obese and give a suitable physical activity guideline taking into account people with different body fat%.

## Background

With the unprecedented economic and social development in China, a host of lifestyle factors have changed daily life dramatically. The prevalence of overweight status increased from 22.8% in 2002 to 30.1% in 2012, with obese increasing from 7.1% in 2002 to 11.9% in 2012 [1, 2]. People who are overweight or obese often face social prejudice, elevated risk of various life threatening diseases including cardiovascular diseases (CVD), diabetes and even cancer, and increased mortality [3–5]. Body mass index (BMI) is regularly used as a robust measure of normal weight, overweight and obese due to its simplicity and the modest correlation with adiposity [6], and its use has been widely recommended in China [7]. However, it is a weak measure of individual fat mass, and healthy persons with high muscle mass can be misclassified as overweight or even obese. Several studies have reported that high body fat percentage (BF%) is an independent risk factor for cardiovascular diseases, coronary events [8–11], and all-cause mortality [12–14]. Physical activity (PA) has been shown to reduce the risk of many of the chronic unhealthy conditions associated with high body fat [15]. However, worldwide around 28% of adults aged 18 years old and above do not meet the WHO-recommended minimum level of PA (150 min per week in moderately to vigorous leisure-time PA) in 2016 [16]. Some evidence suggests a connection between PA and BF%, but previous studies have reported inconsistent results in different age groups. Due to the slowing of metabolism associated with aging [17], an inverse relationship has been observed between PA and BF% in middle-aged adults [18, 19]. However, other studies have observed null relationships among young adults whose average age was 21 years old, possibly due to the weight increasing with fat-free mass rather than the reduction of fat mass [20, 21]. In addition, previous studies used multiple linear regression or categorical logistic regression analyses which can ignore effects limited to a subset of a population and require careful adjustment for non-normal data such as BF% distributions in a population. Based on the inconsistent results from several studies, we propose that quantile regression (QR) is more suitable for analyzing the association between PA and BF%, and that to our knowledge we are the first to do. Therefore, the aims of this study are to (1) quantify the distribution of PA in a large cohort of middle-aged participants of 15 regions in China; (2) describe the distribution of total and trunk BF% at different levels of PA; (3) examine whether the significant association between PA and BF% is uniform across subpopulations after taking BMI into account.

## Methods

### Study design

We leveraged the 2015 wave of the China Health and Nutrition Surveys (CHNS), a large prospective household-based study designed to longitudinally measure how sociological, economic and demographic factors change health and nutritional status across the life span of the Chinese population. The CHNS selected individuals of various ages in ten rounds of surveys from 1989 to 2015, who lived in twelve diverse provinces including Shandong, Liaoning, Heilongjiang, Jiangsu, Henan, Guizhou, Hunan, Hubei, Zhejiang, Yunnan, Shanxi and Guangxi and three autonomous cities including Beijing, Shanghai and Chongqing (in 2011 and 2015). A multi-stage, stratified, random cluster sampling design was used to ensure a balanced representation of urban, suburban and rural areas. Data was derived from the detailed interviewer-administered questionnaires delivered by trained and certified health workers. We had the strict quality control to ensure the data reality, including unified training, the same brand measuring instruments and instruments models etc.

### Study population

For this study we analyzed the 2015 CHNS, which was the first wave to collect body composition data. We limited the sample to participants aged between 40 and 64 years old with complete data on total PA and BF%. Participants with implausible BF% values (< 5%, > 70%, *n* = 75) were excluded from our study. We excluded individuals who were disabled or women who were pregnant or breastfeeding during the survey year (*n* = 74) and subjects who had received diagnoses of hypertension, diabetes, myocardial infarction, stroke, cancer, fracture and asthma (*n* = 1551). Individuals with implausible energy intakes values (< 800 kcal/day or > 6000 kcal/day for men, < 600 kcal/day or > 4000 kcal/day for women, *n* = 236) [22–24] and BMI (< 10 kg/m^2^, > 60 kg/m^2^, *n* = 5) were excluded. Our final analysis sample included 5763 total participants, of which 2540 were men and 3223 were women.

### Anthropometrics

Trained health workers measured weight, height, and BF% (including the total BF%, trunk BF%, and arm and leg BF%). Using a body composition analyzer (BC601, TANITA) weight was measured to the nearest 0.1 kg with the participant standing without shoes and wearing light clothing. Height was measured without shoes to the nearest 0.1 cm using SECA 206 wall-mounted metal tapes. Percentages of body fat were calculated using the bioimpedance method utilizing a proprietary algorithm that requires age, sex, height, and PA level inputs by technicians. This method is being used regularly and has previously been validated in other studies [25, 26]. We recorded the total BF% (ratio of total body fat mass and total body mass) and trunk BF% (ratio of trunk fat mass and trunk mass) separately. As fat distribution is different between men and women, we calculated the ratio of trunk and total BF% to observe the sex disparity. According to a modified BMI for China, we defined overweight and obese based on BMI (weight divided by height in meters squared) cutoff points of 24 and 28 kg/m^2^, respectively. In this study, normal weight group was defined as BMI<24 kg/m^2^ and overweight/obese group was BMI ≥ 24 kg/m^2^.

### Total PA and sedentary behavior assessment

We relied on the standard PA questionnaire, which has been used in our cohort study for nearly 30 years [27], to calculate the average metabolic equivalents of task (MET) hours per day. We defined MET hours as the ratio of a person’s working metabolic rate relative to their resting (basal) metabolic rate. Physical activity was then divided into four domains: travel, occupational, leisure and domestic (e.g. driving, farming, playing basketball, food preparation). For each participant, the average MET·hours per week estimates include both the time spent in each activity and the average intensity of each activity (or sub-activity). We applied the appropriate estimated MET intensity values using the Compendium of Physical Activities based on the lowest level of detail available for each time use survey [28, 29], and aggregated the various subdomain activities to get each individual’s MET·hours per week in each domain. Because there was no international recommended standard categorization for PA, we chose to categorize the total MET·hours per day into Q1, Q2, Q3, Q4 from lowest to highest PA. Details on calculating of how these values have been described in previous literatures [27, 30, 31].

Sedentary behaviors were calculated as the average hours per day (hour/day) spent in various non-occupational recreational activities, including watching TV or movies/videos, reading/writing, playing board games, and using a computer. Time spent engaged in these kinds of activities were summed to obtain total time expenditure on sedentary behaviors, which was not included in the PA calculation.

### Assessment of covariates

Standard questionnaires were used by trained interviewers to collect sociodemographic characteristics, annual per household income, smoking, alcohol consumption, community information (urbanization index) and dietary intake. We categorized the educational level into low, medium and high for primary school education or less, middle school education and high school education and above. Marital status was grouped into two categories (married and single). Participants reported their gross annual per capita household income according to household size, which was inflated to 2015 values and categorized into tertiles. Smoking status was classified as current smoker and not currently smoking. Alcohol consumption refers to whether or not participants have consumed alcohol in the past year.

Energy intake per day and percentage of energy from fat were used as continuous variables, which were collected over a three-day period using a weighing method of condiments in combination with the China Food Composition Table, a validated index of Chinese specific foods and their nutritional contents [22].

Region was grouped as Northern China (Heilongjiang, Liaoning and Beijing), Central China (Shangdong, Jiangsu, Shanghai, Henan and Shanxi) and Southern China (Hubei, Chongqing, Zhejiang, Guizhou, Hunan, Guangxi and Yunnan) based on climate and dietary habit differences. Community urbanization index was calculated based on 12 multidimensional components at the community level reflecting population density, economic activity, traditional markets, modern markets, transportation infrastructure, sanitation, communications, housing, education, diversity, health infrastructure and social services [31, 32], and was categorized into tertiles (high, middle and low). Others details were presented in previous analyses of the CHNS [31].

### Statistical analysis

We calculated descriptive statistics for the individual demographic variables, which were stratified by sex. Continuous variables were expressed as median, the 25th and 75th percentiles. Categorical variables were expressed as percentage. Body fat percentage was described as kernel density diagram by sex and BMI groups. Median (25th, 75th). Physical activity was described by sex and sociodemographic variables and Kruskal-Wallis tests were used for sociodemographic variables. Box plots were used to express the BF% by PA levels, and the difference between each two groups was examined by Kruskal-Wallis tests.

We tested for the interaction because we found that the relationship between PA and BF% might be different for men/women and normal/overweight/obese at the 10th, 25th, 50th, 75th, and 90th percentiles (*p* < 0.0001). So we used separate sex-BMI-stratified QR analyses to assess associations of the total PA, at total body and trunk BF% percentiles. Moderate intensity exercise was defined as 3.0–6.0 MET [33]. We used 4.5 MET·h/d, such as half step jogging [34], to represent the average and tested the association as an additional hour of moderate PA with lower BF%. Compared with the traditional linear regression based on means, QR allows us to evaluate the relationship between PA and BF% at several cut points, which does not require any assumption about the distribution of the regression residuals, and is not influenced by skewness in the distribution of the dependent variable. This method provided greater statistical efficiency when outliers are present and is robust to varying effects of covariates at different percentiles of the response variable [35–37].

We constructed three different models. Model 1 controlled for the total PA only. Model 2 expanded the equation to individual-level variables, such as sedentary activity time, age, educational level, marital status, household income level, energy intake, energy percentage from fat, BMI, smoking status, alcohol consumption status and region. Model 3 expanded the equation further by adding urbanization index level. In all models, the BF% included the total body and the trunk. We considered the statistically significant coefficients by *p* < 0.05. SAS 9.4 (SAS Institute, Inc., USA) was used for all analyses.

## Results

### Demographic characteristics of the study samples

Table 1 displays participant characteristics. Of the 5763 participants (mean age: 52.0 years old) included in the analyses, 1074 (18.6%) lived in northern China and 1892 (32.8%) in central China. In addition, 2802 (48%) were overweight/obese.

**Table 1:** Demographic characteristics among middle-aged Chinese adults in 2015

Variables	Male		Female	
	Normal weight (BMI < 24 kg/m^2^)	Overweight/obese (BMI ≥ 24 kg/m^2^)	Normal weight (BMI < 24 kg/m^2^)	Overweight/obese (BMI ≥ 24 kg/m^2^)
N (number)	1293 (50.9%)	1247 (49.1%)	1668 (51.8%)	1555 (48.2%)
Region (N, %)				
Northern	192 (14.9%)	262 (21.0%)	275 (16.5%)	345 (22.2%)
Central	378 (29.2%)	435 (34.9%)	556 (33.3%)	523 (33.6%)
Southern	723 (55.9%)	550 (44.1%)	837 (50.2%)	687 (44.2%)
Age (year)	53.0 (46.3, 59.3)	50.9 (45.8, 57.0)	51.4 (45.3, 58.1)	51.8 (46.3, 58.1)
Education (N, %)				
Low	444 (38.5%)	517 (44.7%)	524 (38.7%)	426 (33.9%)
Middle	473 (41.0%)	478 (41.3%)	561 (41.5%)	529 (42.1%)
High	237 (20.5%)	163 (14.1%)	268 (19.8%)	302 (24.0%)
Married (N, %)	1223 (94.6%)	1208 (96.9%)	1571 (94.2%)	1460 (93.9%)
Household income (1000 yuan)	15.7 (7.2, 28.0)	18.0 (8.9, 31.8)	16.6 (7.3, 30.0)	15.7 (7.2, 27.9)
Energy intake (1000 kcal/day)	2.1 (1.7, 2.7)	2.2 (1.8, 2.8)	1.8 (1.4, 2.2)	1.8 (1.5, 2.3)
Energy for dietary fat (%)	35.1 (27.1, 44.3)	35.6 (28.2, 43.7)	35.4 (27.7, 43.7)	35.5 (27.6, 43.6)
BMI (kg/m^2^)	21.8 (20.3, 22.9)	26.3 (25.2, 27.9)	22.0 (20.7, 23.0)	26.4 (25.1, 28.2)
Current smoking (N, %)	782 (60.5%)	627 (50.3%)	30 (1.8%)	20 (1.3%)
Alcohol consumption (N, %)	745 (57.6%)	736 (59.0%)	100 (6.0%)	99 (6.4%)
Urbanization Index (score)	67.7 (54.2, 84.2)	74.9 (58.5, 89.3)	73.6 (57.1, 87.0)	73.3 (57.3, 87.2)
Sedentary activity time (hr/week)	15.9 (10.5, 27.0)	16.0 (10.5, 28.0)	14.0 (9.0, 24.5)	14.0 (10.0, 24.5)

Values were expressed as medians (25th, 75th) or number and percentage (N, %)

### The BF% of samples

**Fig. 1:**
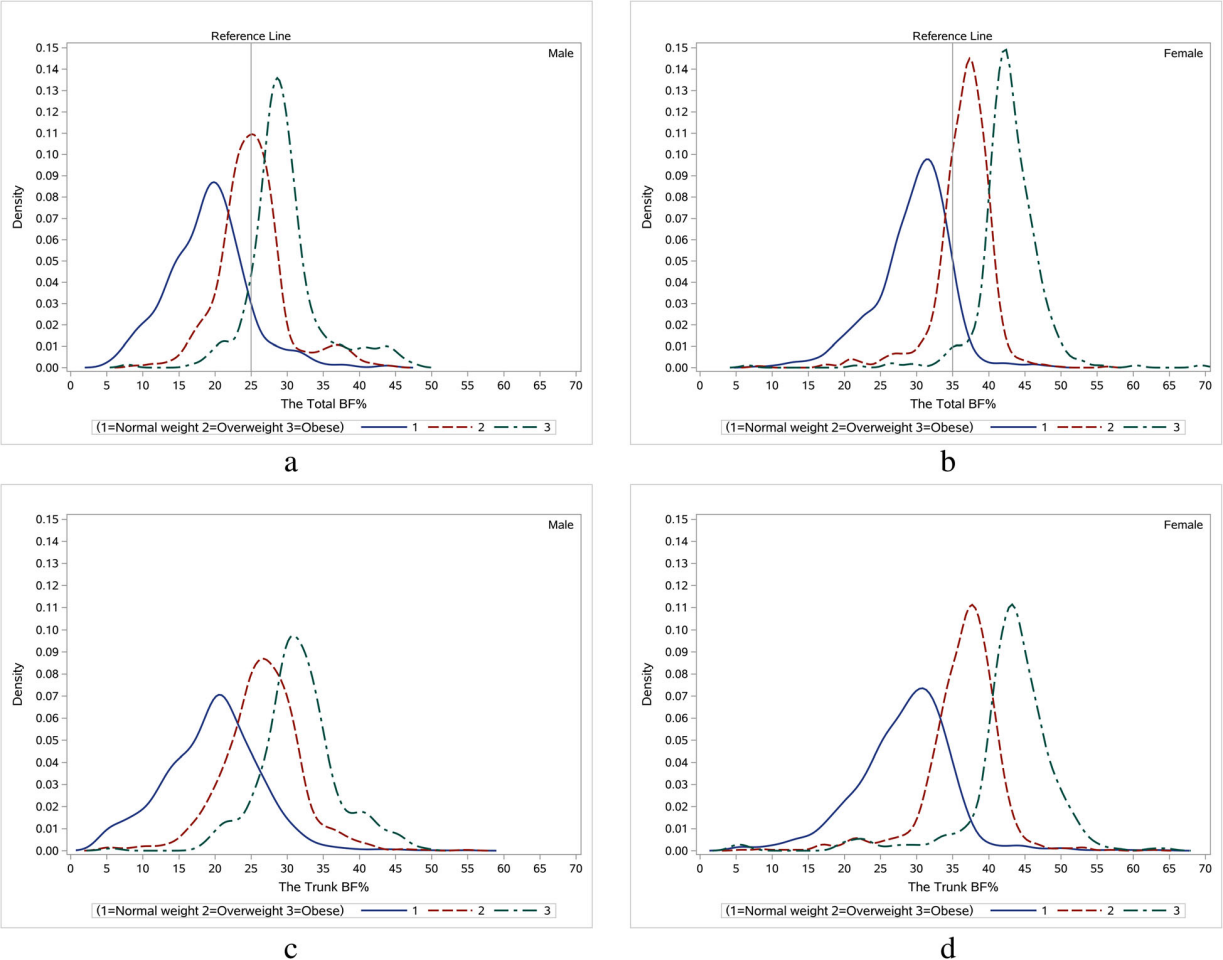
Kernel density diagram for BF% in BMI groups among middle-aged Chinese adults in 2015. Normal weight group, overweight group and obese group were solid line, dash line and dash dot line, respectively. These three groups were divided according to BMI (cutoff points were 24 kg/m^2^ and 28 kg/m^2^). Panel **a** and **b** depicted the total BF% in men and women, respectively. Panel **c** and **d** depicted the trunk BF% in men and women, respectively. The reference line used was the standard (men: 25.0%; women: 35.0%) recommended by AACE/ACE for classifying obese according to the total BF%

Figure 1 shows the smoothed distribution curves of the total BF% and trunk BF% among men and women aged 40–64 years in 2015. Individuals were divided into normal weight group, overweight group and obese group according to BMI. For both males and females, the three curves overlapped with each other, and the overweight curve was roughly between the normal weight curve and the obese curve. The reference line used the standard (men: 25.0%; women: 35.0%) recommended by American Association of Clinical Endocrinology/American College of Endocrinology Obesity Task Force (AACE/ACE) for judging obese according to the total BF% rather than the trunk BF% [38]. Most males in the obese group and a half of males in the overweight group were classified as obese by the standard of the total BF%. However, there were 10.4% of males had normal BMI but high body fat content, which we called normal weight obese [39]. A similar pattern was observed in females, and normal weight obese accounted for 8.8% of normal weight females. The overweight curve and the obese curve in females were further to the right of the reference line than that in males. The patterns in trunk BF% were similar to the total BF%.

### Physical activity by sociodemographic variables

**Fig. 2:**
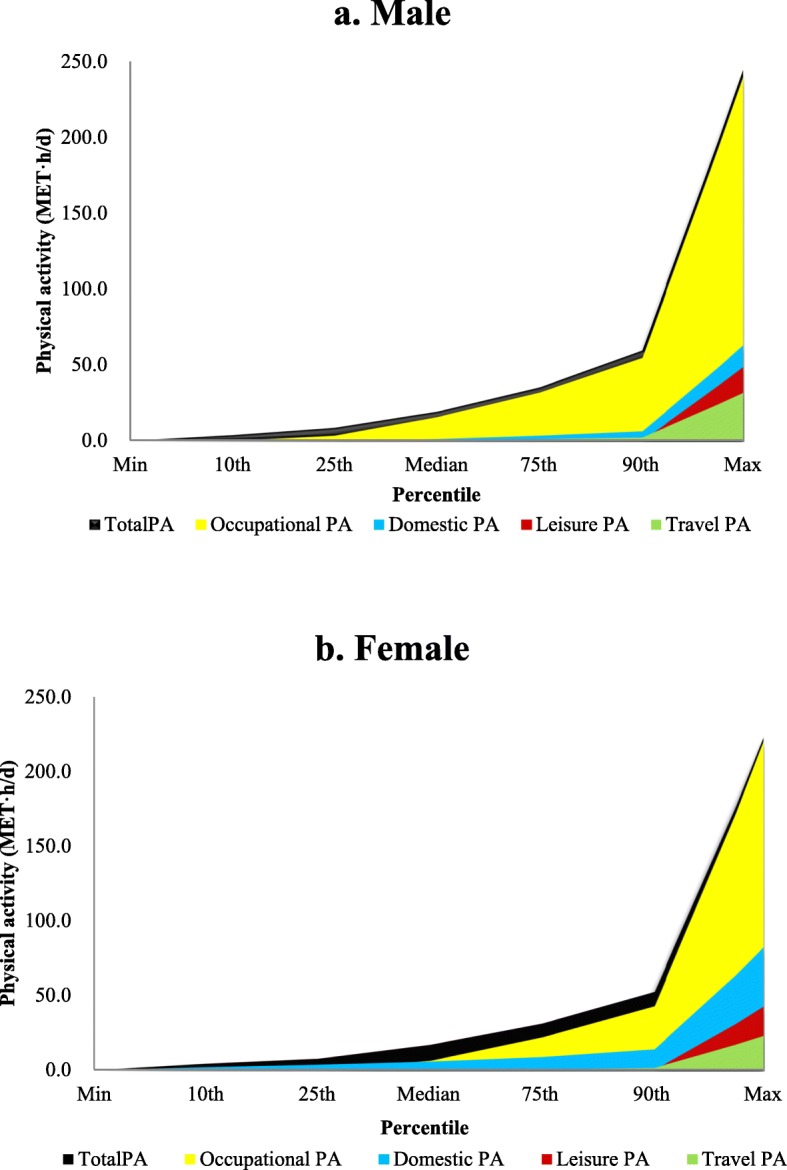
The distribution of total PA and its subdomains among middle-aged Chinese adults in 2015. Panel **a** and **b** depicted the total PA and its subdomains in males and females, respectively

Figure 2 shows the total PA and its four subdomains. The total PA, occupational PA and travel PA in men were statistically higher than that of women (Kruskal-Wallis test, *p* < 0.05), whereas domestic PA and leisure PA were lower in men than women (Kruskal-Wallis test, *p* < 0.05). Occupational PA comprised the main component of total PA and leisure PA was the least. There were only 7.5% of males and 9.9% of females having leisure PA.

Table 2 shows PA by age, BMI groups, education level, household income level, regions and urbanization index level. For both males and females, the median of PA was lower at older ages. PA in females decreased more dramatically than males as age increased, especially between 50-54 y (17.0 MET·h/d) and 55–59 y (11.9 MET·h/d). Men in the overweight/obese group had approximately 13% less PA than men in the normal weight group. The medians of total PA in people with low education were nearly 1.9 times and 1.7 times people with high education in males and females, respectively. People with higher household income had lower PA in both males and females. Women who lived in central China had the lower PA than those in northern or southern China, but no statistical difference were found in region for men. Individuals who lived in low level urbanization areas had nearly two times the amount of PA than those who lived in high level urbanization areas, for both men and women.

**Table 2:** Physical activity by sociodemographic variables among middle-aged Chinese adults in 2015 (MET·h/d)

Sociodemographic Variables	Male		Female	
	N	Median (25th, 75th)	N	Median (25th, 75th)
Age^b^				
40–44	475	20.4 (10.4, 33.7)	594	19.2 (9.1, 33.6)
45–49	460	19.7 (10.0, 36.4)	581	19.9 (9.8, 32.0)
50–54	504	19.3 (8.7, 34.6)	597	17.0 (8.0, 30.4)
55–59	436	17.6 (8.7, 33.8)	413	11.9 (6.8, 25.1)
60–64	361	16.0 (6.0, 30.1)	342	10.2 (6.3, 20.1)
BMI Groups^a^				
Normal weight	1107	20.1 (9.5, 36.9)	1304	16.5 (8.0, 29.5)
Overweight/obese	1129	17.5 (7.8, 31.5)	1223	16.3 (7.6, 29.9)
Education ^a, b^				
High	947	14.9 (7.6, 24.7)	934	13.8 (7.2, 22.4)
Middle	907	21.2 (8.7, 39.5)	1044	17.1 (7.8, 31.3)
Low	382	28.5 (12.9, 48.2)	549	22.9 (9.4, 41.1)
Household Income ^a, b^				
High	807	17.5 (8.8, 29.4)	921	15.7 (7.6, 27.3)
Middle	757	19.3 (8.7, 33.2)	847	17.1 (8.2, 29.7)
Low	672	21.1 (8.5, 40.7)	759	17.1 (7.5, 33.4)
Region^b^				
Northern	429	18.8 (9.1, 38.3)	545	18.0 (8.5, 31.8)
Central	731	18.2 (7.6, 32.4)	857	15.0 (7.0, 28.5)
Southern	1076	19.7 (9.1, 36.2)	1125	17.9 (8.0, 34.3)
Urbanization Index^a, b^				
High	772	13.6 (5.9, 22.8)	1002	11.9 (6.8, 20.9)
Middle	716	18.0 (8.2, 32.4)	859	16.4 (8.0, 29.5)
Low	748	28.7 (14.6, 50.6)	666	27.1 (12.0, 44.4)

^a^*p* < 0.05 in the Kruskal-Wallis test in males. ^b^*p* < 0.05 in the Kruskal-Wallis test in females. ^c^*p* < 0.01 in the Kruskal-Wallis test between sex

### Body fat percentage on different PA levels

**Fig. 3:**
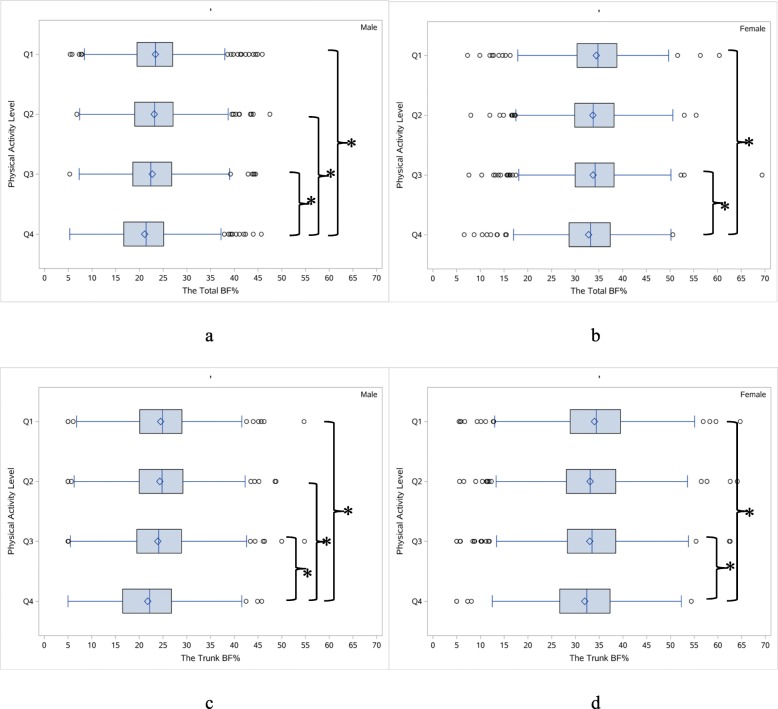
Box plots of the total and trunk BF% among middle-aged Chinese adults in 2015. Panel **a** and **b** denote the total BF% in men and women, respectively. Panel **c** and **d** denote the trunk BF% in men and women, respectively. PA was grouped into Q1, Q2, Q3, Q4 by ascending order according to the interquartile. * indicates a significant difference between the two groups

The shape of the total and trunk BF% distribution differed across the levels of PA with respect to location, spread, and skewness (Fig. 3). Male participants in the highest quartile of PA had a median total BF% of 21.4%, which was statistically significantly lower than the other three PA groups (Q1: 23.4%, Q2: 23.0%, Q3: 22.4%) (*p* < 0.05), but no statistically significant differences were observed when comparing Q2, Q3 and Q4 groups with each other. Female participants who engaged in the highest quartile of PA had a median total BF% of 33.3%, which was statistically significantly lower than Q1 (34.8%) and Q3 group (34.2%) (*p* < 0.05). The trunk BF% of males in the highest quartile of PA (22.2%) was significantly lower than the other three groups (Q1: 24.9%, Q2: 24.8%, Q3: 24.1%) (*p* < 0.05), while BF% of females in the highest quartile of PA (32.4%) were also significantly lower than Q1 (34.4%) and Q3 (33.5%) groups. The ratio of trunk and total BF% represented the sex disparity in fat distribution, which increased quickly and dramatically in men (see Additional file 1).

### The association between PA in different levels and BF% at different percentiles

Table 3 illustrates that the total BF% and the trunk BF% at the 10th, 25th, 50th, 75th and 90th percentiles changed with 4.5 MET·h/d among men and women. We used 4.5 MET·h/d to represent the average level of the moderate intensity exercise because this was the recommendation value in China. Overall, 1 hour extra moderate PA (4.5 MET·h/d) was significantly associated with 0.029, 0.054, and 0.070% less total BF% at the 50th, 75th and 90th percentiles respectively, and 0.049, 0.086, and 0.099% less trunk fat at the same percentiles respectively (see Additional file 2). In normal weight and overweight/obese groups of both males and females, BF% was significantly inversely associated with PA at some special percentiles. The results obtained with Model 1 suggested that there were significant inverse relationships between PA and BF% at most of the percentiles. However, the negative significant coefficients changed at different percentiles after adjusting for individual level and community level factors.

**Table 3:** The sex-BMI-stratified coefficients of PA on BF% among middle-aged Chinese adults in 2015

Variables		Normal weight (BMI < 24 kg/m^2^)					Overweight/obese (BMI ≥ 24 kg/m^2^)				
		10th	25th	50th	75th	90th	10th	25th	50th	75th	90th
Total BF%											
Male	Model 1	−0.075	− 0.150^*^	− 0.105^*^	− 0.085^*^	− 0.111^*^	− 0.079	− 0.084^*^	− 0.103^*^	− 0.068^*^	− 0.034
	Model 2	−0.004	− 0.027	− 0.060^*^	− 0.079^*^	− 0.091^*^	− 0.101	− 0.069^*^	− 0.045^*^	− 0.002	− 0.054
	Model 3	0.020	0.003	− 0.032	− 0.079^*^	− 0.110^*^	− 0.098	− 0.071^*^	−0.032	0.014	−0.052
Female	Model 1	−0.119^*^	− 0.110^*^	− 0.081^*^	− 0.077^*^	− 0.082^*^	− 0.061	− 0.043	−0.049^*^	− 0.082^*^	− 0.123^*^
	Model 2	0.011	−0.049^*^	− 0.059^*^	− 0.044	− 0.063^*^	− 0.002	− 0.021^*^	− 0.035^*^	−0.032^*^	− 0.031^*^
	Model 3	0.046	−0.046^*^	− 0.073^*^	− 0.057^*^	− 0.098^*^	− 0.006	−0.023	− 0.035^*^	−0.039^*^	− 0.037^*^
Trunk BF%											
Male	Model 1	− 0.133^*^	− 0.181^*^	− 0.130^*^	− 0.127^*^	− 0.152^*^	− 0.135^*^	− 0.157^*^	− 0.124^*^	− 0.078^*^	0.031
	Model 2	−0.054	− 0.044^*^	− 0.086^*^	− 0.098^*^	− 0.137^*^	− 0.128	− 0.078	− 0.061^*^	0.007	− 0.018
	Model 3	0.004	− 0.029	− 0.100^*^	− 0.104^*^	− 0.173^*^	− 0.138	− 0.076	− 0.054	0.020	− 0.020
Female	Model 1	−0.076	−0.131^*^	− 0.115^*^	−0.109^*^	− 0.104^*^	−0.127	− 0.060	−0.065^*^	− 0.114^*^	−0.138^*^
	Model 2	−0.116^*^	−0.097^*^	− 0.067^*^	−0.071^*^	− 0.081^*^	−0.046	− 0.038	−0.056^*^	− 0.047^*^	−0.055^*^
	Model 3	−0.039	−0.124^*^	− 0.108^*^	−0.079^*^	− 0.083	−0.040	− 0.036	−0.059^*^	− 0.050^*^	−0.053^*^

The coefficients were based on the quantile regression at 10th, 25th, 50th, 75th, and 90th percentiles. ^*^p < 0.05. (*P* values see Additional file 4.) Model 1 was controlled for the total PA only. The individual-level variables were then added to the equation to estimate Model 2, including the sedentary activity time, age, educational level, marital status, household income level, energy intake, energy percentage from fat, BMI, smoking status, alcohol consumption status and region. For Model 3, urbanization index level was added to Model 2

The results obtained with Model 3 were different between men and women. First, females had more statistical significant coefficients at multiple percentiles than males. Specifically, there were four significant coefficients of total BF% in normal weight women, which appeared at the 25th, 50th, 75th, and 90th percentiles, respectively, but only two in men, appearing at the 75th and 90th percentiles, respectively. Second, for overweight/obese group, significant coefficients appeared in women at the upper percentiles (50th, 75th and 90th), but in men at only the 25th percentile. Third, the associations between PA and total BF% were weaker in females than in males.

We found the absolute coefficients in overweight/obese group were lower than that in normal weight group. The significant coefficients were at the upper percentiles (75th and 90th) in normal weight men, and at the lower percentiles (25th) in overweight/obese men. The percentiles where the significant regression coefficients appeared corresponded with the range of 22–25% in male BF% (see Table 3 and Additional file 3). The significant coefficients from the 25th to the 90th percentiles in normal weight women were approximately twice that of overweight/obese women from the 50th to the 90th percentiles. The absolute coefficients in trunk were bigger than that in total body in the corresponding percentiles. The significant coefficients appeared in the normal weight males from 50th to 90th percentiles and 25th to 75th percentiles in females. The coefficients in overweight/obese were significant from the 50th to 90th percentiles in females but not across any of the percentiles in males.

## Discussion

Our analysis of middle-aged adults from the 2015 wave of CHNS, PA showed marked sociodemographic variations in PA levels. In addition, it found inverse associations between PA levels and BF%, similar to previous studies conducted in China [18], Indian [21] and Southern Tasmania [40] though the different measurement. Occupational PA comprised the greatest portion of the total PA in China [31], especially among farmers in rural areas or construction workers in urban areas. These individuals had a much higher total PA than those work in an office setting. Leisure-time PA appeared to be much lower in Chinese than the levels described in western populations, such as the United States and the United Kingdom [27]. Domestic PA in women was little higher than that in men, tracking with the societal norms where women still undertake most of the housework. Total PA decreased dramatically with aging, and was closely tied to social and economic development. The main sociodemographic factors associated with lower total PA aging, high level of education and income, living region in central China and high level of urbanization index. These findings were consistent with a previous study by Du et al. [18]. Therefore, these variables that distinctly changed by PA, as well as lifestyle habits represented by smoking and drinking, and individual energy-intake and ratio of energy from dietary fat, should be considered in the QR model.

An additional hour of moderate PA was associated with lower total BF% in several percentiles instead of across all the percentiles. The results were analyzed by QR method, which allowed the skewness distribution and the outliers that we could not exclude by judging the rationality. There were differences by sex in the relationship between PA and BF%, that might be due to the physiological and basal metabolic rate differences between males and females. The inverse relationship between PA and the total BF% was significant for women at most percentiles of BF% distribution (approximately from 27.0 to 44.0%), while it appeared only at the total BF% around 22 to 25% in men. An extra hour of moderate exercise per day might not be enough to reduce fat mass for a man with BF% over 25% (overweight/obese men at the 50th, 75th and 90th percentiles) and it might increase the fat-free mass rather than decrease fat mass for a man with healthy BF% (under 20%, normal weight men at the 10th to 50th percentiles) [33]. The absolute significant coefficients in overweight/obese women at the 50th to 90th percentiles were about half of normal weight (see Table 3). These findings suggest that overweight/obese women might increase the intensity and duration of vigorous exercise to achieve the aim of BF% reduction.

Our results are consistent with prior studies have reported that PA was associated with diminished body fat mass. However, we were unable to compare the strength of associations with adiposity observed in our study directly with those previously observed because of differences in the study design. Du reported that greater PA (14 MET·h/d) was associated with 0.48 (95% CI: 0.45, 0.50) percentage points less body fat in 30–79 years old, using the interviewer-administered PA questionnaire and BIA, from the China Kadoorie Biobank study and employing the multiple linear and logistic regression models [18]. Bradbury reported that compared with < 5 excess MET·h/week at baseline, ≥100 excess MET·h/week was associated with a 2.8 percentage points lower body fat in men and 4.0 percentage points lower body fat in women aged 40–69 years in UK, using International Physical Activity Questionnaire and BIA and employing the multiple linear regression model [19]. Bowen reported that one MET·h higher activity was associated with 145 g less body fat (95% CI 73,218) in the middle-aged Indian, using PA questionnaire and dual energy X-ray absorptiometry (DXA) scans and employing the multiple linear regression model [21].

Moreover, an extra hour per day moderate PA had inverse association with trunk BF%, and the effect was more pronounced than the total BF%. We found that it was more common for men to gain fat in the abdomen (android), most of which was visceral adipose rather than subcutaneous adipose, whereas women store more fat in the gluteal-femoral region (gynoid) [41]. As body weight increased, the ratio of trunk and total BF% rose dramatically in men (see Additional file 1). Min reported that regional fat distribution in the android and gynoid regions had different effects on lipid profiles [42], and fat in the android region may be an important factor in determining the risk of CVD [41]. It was reported that men had a higher standardized mortality rate of CVD than women in China from 1990 to 2013, and android fat maybe one of the main contributors for men [2].

In general, overweight/obese group showed a higher BF% than normal weight group, however we did see an interaction between the BF% density curves of the two groups. About 10% of normal weight individuals had high body fat. Several studies discuss the term “normal weight obese”, which describes an individual with normal body weight and BMI (< 24 kg/m^2^) but high BF%, accompanied with total lean mass deficiency [8, 39, 43, 44]. Some studies have reported associations with normal weight obese and a high prevalence of cardiometabolic dysregulation, metabolic syndrome, and CV risk factors [39, 45]. In our study, normal weight obese subjects were distributed at the 90th percentile and above the total BF% of the normal weight group (see Additional file 3). In addition, an extra hour per day of moderate PA was more strongly and inversely associated with the total BF% for the normal weight obesity subjects than others (see Table 3). For example, in normal weight at the 90th percentiles, an extra 4.5 MET·h/d PA was associated with 0.110 and 0.098 percentage points lower total BF% for men and women respectively, and these relationships were stronger than that at the 10th, 25th, 50th, and 75th percentiles. Half of overweight men and most of overweight women were classified obese according to the standard of BF% (see Fig. 1), which could indicate that overweight men are more likely to gain muscle, while overweight women are more likely to gain fat.

To all segments of the population, health and functional benefits are visible from PA [31, 46, 47]. Many countries have published guidelines to encourage increase of PA levels, including the USA, Canada, and China [36]. All of these guidelines follow the same recommendations as the World Health Organization, which states that 150 min/week (7.5 MET·h/w) in leisure-time PA is the minimal level to reduce risk of non-communicable diseases, and 300 min/week (15 MET·h/w) is the level to maintain weight [48]. However, guidance is general and not personalized for individuals with different BMI and BF%, especially for normal weight obese. There is also no suitable guideline on PA for different levels of overweight or obese adults. As the fact of only about 10 % population having leisure-time PA (see Fig. 2), it might be more effective if PA guideline was personalized in a way that took into account people with different body fat according to statistically significant percentiles.

This study has several limitations. First, the anthropometric methods we used to measure PA and BF% in our study were not the most precise available, but they were broadly acceptable and were the most economical and useful way for a large-scale survey covering 15 regions in China. In this 30-year large scale follow-up survey, it was impractical to obtain objective measures of usual activity level using accelerometer and BF% measured by DXA. In our study, we used an interviewer-administered PA questionnaire, about frequency, duration, and intensity of PA within 1 week in several different domains, which had been tested, modified, and used from 1989. Although data on PA was accumulated from recall might be prone to overestimation and the interview progress seemed complex because of a large number of PA question [20], we chose this approach because the questionnaire was revised from the international PA questionnaire and we have remained to use it consistently with each wave of the CHNS. Our questionnaire was effective and validated by professors from the University of North Carolina at Chapel Hill, as well as the National Institute for Nutrition and Health, Chinese Center for Disease Control and Prevention. Bioelectrical impedance analysis was more suitable and reasonable to measure the BF% in this large scale field investigation because of its cost-efficiency and easy-carried [6].

Second, though CHNS was a long-term follow-up study, 2015 was the first year to collect body composition data. However, we would continue extend this data collection to future surveys in order to validate the causal relationship between PA and BF%. Third, because the subjects in our study were middle aged people with similar demographic characteristics, it may be difficult to generalize our results to other populations. Although these limitations existed, our study was based on a large scale field survey comprising a lot of provinces, and our strict quality control ensured our accurate results, which imparted the ability to make certain generalizations.

## Conclusions

In this sample of middle-aged Chinese adults, we found that individuals who were doing the most PA had a lower BF% than those who were doing the least PA. Older adults, overweight/obese group, higher education, higher income, living in central China and higher urbanization were the sociodemographic factors associated with lower PA. However, we found this inverse association between PA and BF% was only in particular subpopulations rather than the entire population, specific for the normal weight men with BF% at 22.0–25.0%, the overweight/obese men with 23.1%, normal weight women with 27.0–34.7%, and overweight/obese women with 38.1–44.0%. We should pay much attention to normal weight obese and give a suitable PA guideline taking into account people with different BF%.

## Supplementary information

**Additional file 1. **Ratios of trunk and total body fat% at the 10th, 25th, 50th, 75th and 90th percentiles. * *p* < 0.05. The overall coefficients were based on the quantile regression in Model 3. Model 3 adjusted the sedentary activity time, age, educational level, marital status, household income level, energy intake, energy percentage from fat, BMI, smoking status, alcohol consumption status, region and urbanization index.

**Additional file 2.** The overall coefficients of physical activity on body fat% among middle-aged Chinese adults in 2015.

**Additional file 3.** Body fat% distribution among middle-aged Chinese adults in 2015.

**Additional file 4. **The sex-BMI-stratified coefficients of PA on BF% among middle-aged Chinese adults in 2015. The coefficients were based on the quantile regression at 10th, 25th, 50th, 75th, and 90th percentiles. * *p* < 0.05. Model 1 was controlled for the total PA only. The individual-level variables were then added to the equation to estimate Model 2, including the sedentary activity time, age, educational level, marital status, household income level, energy intake, energy percentage from fat, BMI, smoking status, alcohol consumption status and region. For Model 3, urbanization index level was add to Model 2. Additional file 4 is a supplement file providing *p* values for Table 3.

## Data Availability

Our study relied on data from China Health and Nutrition Surveys. The datasets using during the current study are available from the corresponding author on reasonable request.

## Abbreviations

BF%: Body fat percentage
PA: Physical activity
BMI: Body mass index
CVD: Cardiovascular diseases
CHNS: China Health and Nutrition Survey
QR: Quantile regression
DXA: Dual energy x-ray absorptiometry
BIA: Bioelectrical impedance analysis
