# Supplementary material for: The association between physical activity and body fat percentage with adjustment for body mass index among middle-aged adults: China health and nutrition survey in 2015
Source: BMC Public Health. 2020 May 19;20:732. doi: 10.1186/s12889-020-08832-0 (PMC7238529; doi:10.1186/s12889-020-08832-0)
Supplement: Supplementary file 1 — Additional file 1. Ratios of trunk and total body fat% at the 10th, 25th, 50th, 75th and 90th percentiles. * p < 0.05. The overall coefficients were based on the quantile regression in Model 3. Model 3 adjusted the sedentary activity time, age, educational level, marital status, household income level, energy intake, energy percentage from fat, BMI, smoking status, alcohol consumption status, region and urbanization index. [file 12889_2020_8832_MOESM1_ESM.pdf]

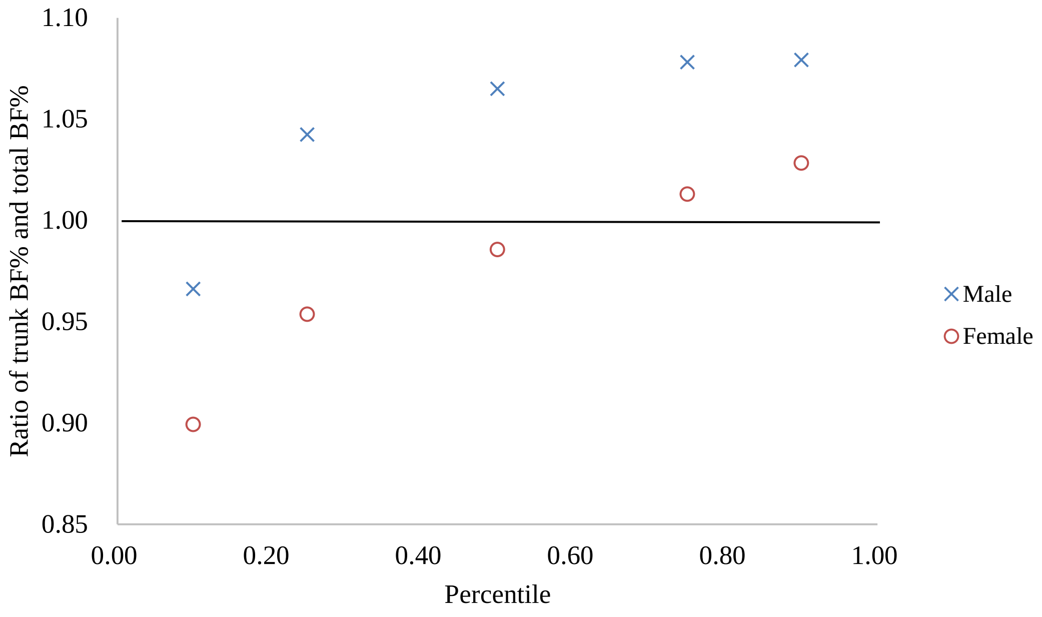

Additional File 1. Ratios of trunk and total body fat% at the 10th, 25th, 50th, 75th and 90th percentiles.
